# THE ROLE OF VERBATIM VERSUS GIST FORMATTING IN AGE-RELATED DIFFERENCES IN INFORMATION AVOIDANCE

**DOI:** 10.1093/geroni/igad104.2632

**Published:** 2023-12-21

**Authors:** Lauren Mei, Julia Nolte, Corinna Löckenhoff

**Affiliations:** Weill Cornell Medicine, New York City, New York, United States; Tilburg University, Tilburg, Noord-Brabant, Netherlands; Cornell University, Ithaca, New York, United States

## Abstract

Older adults are more likely to underuse and actively avoid decision-relevant information. Although the mechanisms underlying age-related differences in information avoidance are still unknown, Fuzzy-Trace Theory (Reyna, 2012) suggests they may be due to age-related differences in information processing preferences: According to this framework, information is stored using both gist (subjective meaning) and verbatim representations (objective details/numbers). Older adults prefer gist information and gist-based information processing, whereas younger adults favor verbatim information and verbatim processing. To examine whether age-related differences in information avoidance are affected by age-related information and processing preferences, an adult lifespan sample (N = 432) completed a pre-registered online study. Participants responded to scenarios containing potentially aversive information and chose between accepting or avoiding the information. Information formatting was experimentally manipulated, with participants either being faced with receiving gist or verbatim-formatted information. Across age groups, participants were more likely to avoid verbatim numbers than qualitative gist information (p=.026); no associations were found between avoidance and information or processing preferences. At odds with prior research, age was associated with lower information avoidance (p=.010). Follow-up analyses revealed that older adults were less likely to avoid information in health-related contexts but not in contexts unrelated to health (e.g., consumer information; p=.010). Overall, participants were more likely to avoid non-health information than health information (p<.001). In sum, our findings revealed that older adults show a decreased likelihood of avoiding potentially aversive health information, but that age-related differences in information avoidance cannot be linked to age-related information or processing preferences.

